# DN4‐Positive Neuropathic Pain Features in End‐Stage Knee Osteoarthritis Awaiting Arthroplasty: Prevalence and Relationship With Function and Quality‐of‐Life

**DOI:** 10.1111/1756-185x.70882

**Published:** 2026-09-22

**Authors:** Alan P. Mozella, Ana Carolina Leal, Rafael Erthal de Paula, Aline Cordeiro Fernandes Ladeira, Sandra Minamoto, Matheus Carvalho, Jordanna Franco Sucupira, Ricardo Kobayashi, Thiago Lemos

**Affiliations:** ^1^ Centro de Cirurgia Do Joelho Instituto Nacional de Traumatologia e Ortopedia – INTO Rio de Janeiro Brazil; ^2^ Faculdade de Ciências Médicas Universidade do Estado do Rio de Janeiro – UERJ Rio de Janeiro Brazil; ^3^ Divisão de Ensino e Pesquisa Instituto Nacional de Traumatologia e Ortopedia – INTO Rio de Janeiro Brazil; ^4^ Centro de Dor Universidade de São Paulo – USP São Paulo Brazil

**Keywords:** health‐related quality of life, musculoskeletal diseases, neuralgia, orthopedic procedures, pain measurement

## Abstract

**Objectives:**

This cross‐sectional study investigated the occurrence of neuropathic pain features (NP) in individuals with end‐stage knee osteoarthritis (KOA) referred to total knee arthroplasty (TKA) and explored its association with functional and quality‐of‐life outcomes.

**Methods:**

In 902 patients, NP was screened using the Douleur Neuropathique 4 (scores ≥ 4). Distribution of NP features (positive/negative) regarding sex were assessed with *z*‐test for proportions. The influence of NP features on the outcomes (visual analog scale for pain, WOMAC, SF‐36) were tested with the Mann–Whitney *U*, followed by a multivariate logistic regression model to identify independent predictors of NP features, using 5% as statistical threshold.

**Results:**

The cohort was 72.6% female, with a mean ± SD age of 70 ± 8 years. NP‐positive features prevalence was 28.6% (95% CI of 25.7%–31.6%), which was significantly higher in females (31.5%) than males (21.1%; *p* < 0.001). Patients with NP‐positive features reported worse scores across all measured domains: higher pain intensity (VAS; *p* < 0.001), worse knee‐related symptoms/function (WOMAC pain, function, and total scores; all *p* < 0.001), and lower quality‐of‐life in specific SF‐36 domains (general health, vitality, role‐emotional, mental health; all *p* < 0.01). The multivariate model confirmed that higher WOMAC total score and specific SF‐36 scores were independently associated with NP‐positive features.

**Conclusions:**

NP features are prevalent in approximately one‐third of the patients with end‐stage KOA awaiting arthroplasty, particularly among females. Its presence is independently associated with poorer joint‐related function and reduced quality‐of‐life, suggesting the potential value of NP features assessment and management into pre‐ and postsurgical care.

## 1 Introduction

Knee osteoarthritis (KOA) is a prevalent chronic disease characterized by the degradation of hyaline cartilage, accompanied by various metabolic alterations and structural damage in different joint tissues. Local joint pain, one of the main symptoms of KOA [1], is often identified as nociceptive or inflammatory in nature [1]. However, the neuropathic component of KOA pain has long been identified [2, 3].

Neuropathic pain results from complex changes in the nervous system, altering responses to pain stimuli [4]. Common manifestations, such as central sensitization and hyperalgesia, often occur due to abnormal activation of nociceptive pathways, persisting beyond the initial stimulus [5]. Additionally, neuropathic pain includes symptoms like paresthesia, allodynia, and numbness, complicating diagnosis [6].

Recent evidence indicates that the prevalence of neuropathic pain in KOA patients ranges from 0.5% to 52% [2, 7, 8, 9, 10, 11, 12, 13], depending on the patients' clinical status. However, it is important to acknowledge that this wide reported prevalence range reflects not only differences in patients' clinical status but also disparities in the diagnostic criteria and tools used across studies. This methodological heterogeneity is a significant limitation. This limitation is particularly relevant in the end‐stage KOA population referred for total knee arthroplasty (TKA), for which evidence is scarce, particularly with regard to joint function, clinical conditions, and quality‐of‐life outcomes [3, 14, 15]. This evidence gap hinders the development of individualized clinical management strategies [16].

To address this, various questionnaires, such as the *Douleur Neuropathique en 4 Questions* (DN4 [17, 18]), are used in clinical and research settings. The DN4 questionnaire is a well‐validated, standardized tool widely recommended for screening neuropathic pain in both clinical and research settings [19]. Its application is common in KOA research, which facilitates comparison across studies [20]. Its practical advantages include brevity and the inclusion of a brief sensory examination, which adds an objective clinical dimension.

DN4 has demonstrated strong validity and reliability. A recent systematic review of 22 studies indicated it has good diagnostic accuracy (82% sensitivity and 76% specificity), and an excellent overall discriminative ability (area under the curve of 0.89) [19]. Designed to be a screening instrument and not a definitive diagnostic tool, DN4 serves to identify patients with neuropathic pain features; however, the potential confounding influence of coexisting nociceptive or inflammatory pain must be acknowledged.

This study aimed to investigate the prevalence of neuropathic pain features (DN4‐positive, scores ≥ 4) in patients with end‐stage KOA referred to TKA and to explore its association with joint function and quality‐of‐life measures. Pain was broadly assessed not only using the DN4 but also with instruments that account for different components of pain, such as Visual Analog Scale and the Western Ontario and McMaster Universities Osteoarthritis Index (WOMAC) pain domain.

## 2 Methods

### 2.1 Participants and Ethical Concerns

This cross‐sectional study was approved by the local ethical committee (process no. 39785020.7.0000.5273, approved on December 14th, 2020). It included patients with severe knee osteoarthritis (KOA) awaiting total knee arthroplasty (TKA). The osteoarthritis stage was determined through radiographic examinations (anterior–posterior view with load, lateral, and axial views of the patella at 30°), and classified according to Kelgren Lawrence and Ahlbäck criteria [21]. Exclusions were refusal to consent or incomplete questionnaires.

**FIGURE 1:**
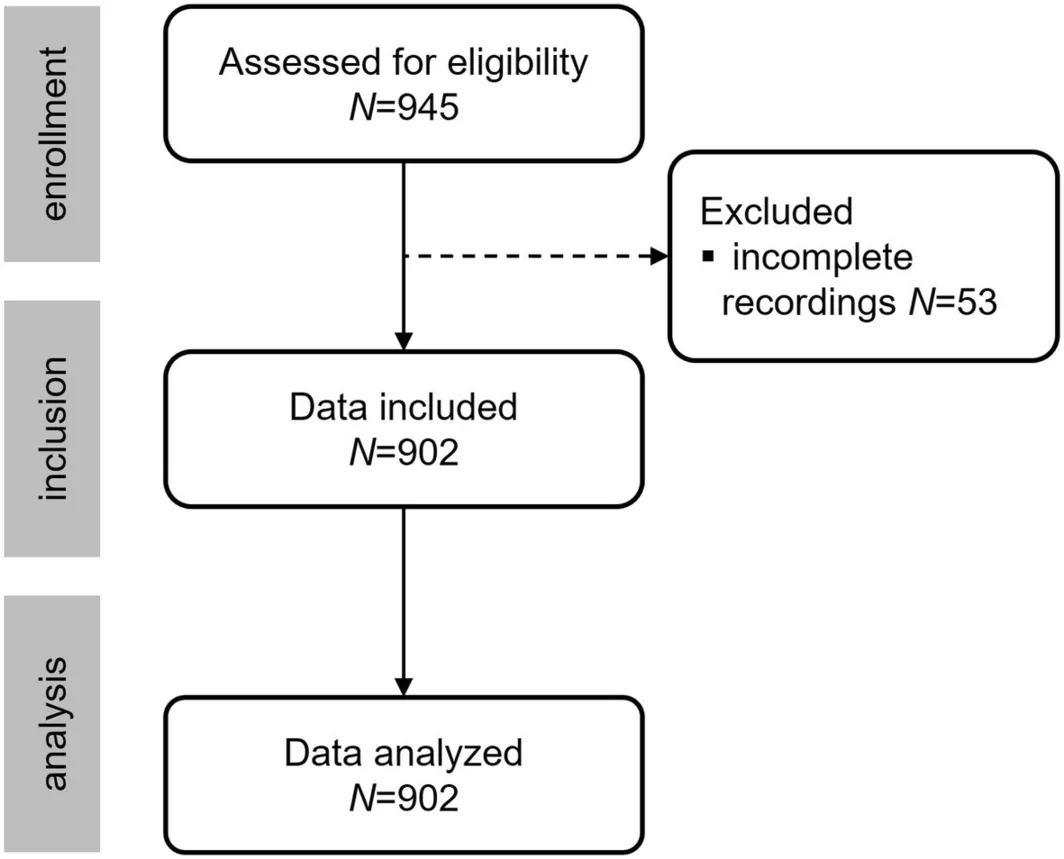
Flowchart of sample selection and allocation.

Data was collected from June 2019 to January 2021. From 945 eligible patients, 902 (aged between 36 and 95 years old; 70 ± 8 years; mean ± SD) were evaluated (Figure 1). Forty‐three patients were excluded due to incomplete data.

### 2.2 Assessments

All assessments were performed during the preoperative period, specifically on the day before surgery. Demographic data and pain sensation using the visual analog scale (VAS) were collected. The presence of neuropathic pain features was assessed with the *Douleur Neuropathique en 4 Questions* (DN4). The questionnaire contains 10 yes/no items related to symptoms and signs of neuropathic pain, and a cutoff value of 4 or higher is used to identify individuals with positive indications of this condition [17, 18].

Additionally, further instruments were also applied. The Knee Society Score (KSS) evaluates the outcomes of total knee arthroplasty and total knee replacement surgeries through objective and functional rating systems [22]. The Western Ontario and McMaster Universities Osteoarthritis Index (WOMAC) assesses the severity of osteoarthritis, covering pain, stiffness, and physical function domains [23]. Finally, the Medical Outcomes Study Short Form‐36 questionnaire (SF‐36) measures self‐reported health‐related quality‐of‐life in eight domains: physical functioning; role limitations due to physical health problems; bodily pain; general health; vitality; social functioning; role limitations due to emotional problems; and mental health [24].

### 2.3 Statistical Analysis

All analysis was performed in Python (v. 3.11.5) and JASP (v. 0.18.1). Neuropathic pain features (NP) prevalence was calculated for the total sample and each sex, with proportions compared using a *z*‐test of proportions.

As data significantly deviated from normality (Shapiro–Wilk, all *p* < 0.011), nonparametric tests were used. The Mann–Whitney *U* test compared outcomes between NP‐positive and NP‐negative groups. The effect size was estimated with point‐biserial correlation coefficient (*r*_BS_). The statistical threshold was set at 5% and adjusted for multiple comparisons using the False Discovery Rate method when necessary.

A multivariate logistic regression was used to identify factors (joint function and quality‐of‐life measures) associated with the distribution of NP‐positive features, excluding redundant pain‐related variables (e.g., VAS and the pain scores of the WOMAC and SF‐36). The backward stepwise regression method was used for variable selection: initially, all predictor variables of interest (KSS, WOMAC domains and SF‐36 domains) were included in the model; subsequently, variables were removed one by one, using *p* > 0.05 in the Wald test as the exclusion criterion. The process was repeated until all remaining variables in the model showed *p* < 0.05. The performance of the final model was evaluated using the area under the ROC curve (AUC) and the classification matrix, employing the standard cutoff point of 0.5.

## 3 Results

### 3.1 Sample Description, Stratified by Sex

When stratified by sex, the cohort comprised 655 females (72.6%) and 247 males (27.4%), with a significant difference in sex proportion (*p* < 0.001). Median (1st–3rd quartiles) age was similar between females and males [69 (65–75) vs. 68 (63–75) years; *p* = 0.057, *r*_BS_ = 0.082]. Visual Analog Scale (VAS) scores were significantly higher in females compared to males [9 (8–10) vs. 8 (7–9); *p* < 0.001, *r*_BS_ = 0.298], indicating greater pain intensity among female patients.

### 3.2 Prevalence of DN4‐Positive Neuropathic Pain Features in Patients Undergoing TKA

**FIGURE 2:**
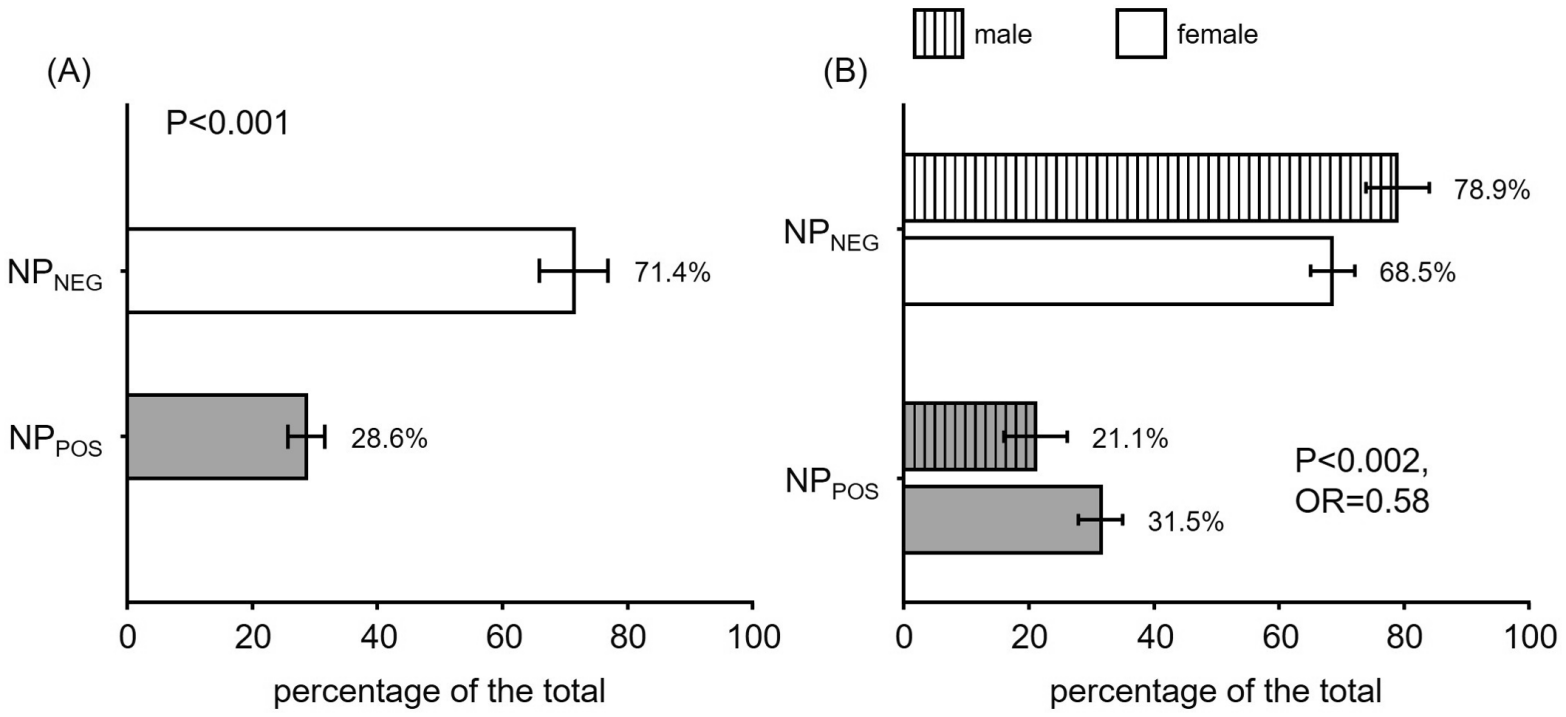
Prevalence of DN4‐positive neuropathic pain features (NP_POS_, positive; NP_NEG_, negative): (A) overall proportion of participants with NP_NEG_ and NP_POS_; (B) proportion of NP_NEG_ and NP_POS_ among females and males. Bars represent the proportion estimates, with error bars denoting the 95% confidence intervals (white bars, NP_NEG_; gray bars, NP_POS_; dashed bars in B, proportion for males). The *p*‐value in (A) corresponds to the proportion test comparing the overall prevalence of NP_NEG_ and NP_POS_, while the *p*‐value in (B) refers to the proportion test comparing the prevalence of NP_POS_ between the sexes. The odds ratio (OR) for neuropathic pain in males relative to females (reference) is also shown.

Among the 902 patients evaluated, DN4‐positive neuropathic pain features (NP‐positive) were present in 258 patients, while the remaining 644 were NP‐negative, with significant differences between the proportions (*p* < 0.001; Figure 2A). The estimated prevalence of NP‐positive features in this cohort of patients with end‐stage KOA awaiting TKA was 28.6% (95% CI of 25.7%–31.6%). There was a higher prevalence of NP‐positive features among females [31.5% (27.9%–35.0%)] compared to males [21.1% (16.0%–26.1%); *p* = 0.002; Figure 2B]. Using females as the reference category, the odds ratio for NP‐positive features in males was 0.58 (95% CI 0.40–0.83), indicating approximately 42% lower odds of NP‐positive features in males compared with females.

### 3.3 Differences in Functional and Quality‐Of‐Life Outcomes Between Patient Groups

Descriptive statistics and between‐group comparisons are presented in Table 1. Age, Knee Society Scores and range of motion did not differ between groups (adjusted P always > 0.119). The NP‐positive feature group reported significantly higher scores on the Visual Analog Scale (9.0 vs. 8.0 [medians], *p* < 0.001, *r*_BS_ = −0.223) and all WOMAC domains (pain, 14 vs. 13, *p* < 0.001, *r*_BS_ = −0.155; function, 51 vs. 48, *p* < 0.001, *r*_BS_ = −0.165; total score, 69 vs. 65, *p* < 0.001, *r*_BS_ = −0.171), except for WOMAC stiffness (5 vs. 5, *p* = 0.002, *r*_BS_ = −0.142), which showed no clear direction of difference. Regarding SF‐36, NP‐positive feature patients scored lower on general health (45 vs. 50, *p* = 0.008, *r*_BS_ = 0.118), vitality (45 vs. 50, *p* < 0.001, *r*_BS_ = 0.179), mental health (56 vs. 64, *p* < 0.001, *r*_BS_ = 0.227). while emotional role limitations showed the same median in both groups (0 vs. 0), although the interquartile range (0–67 vs. 0–33) indicates a shift toward worse scores in the NP‐positive feature group (*p* < 0.001, *r*_BS_ = 0.162). Although statistically significant, physical functioning (10 vs. 10, *p* = 0.028, *r*_BS_ = 0.099) and physical role limitations (0 vs. 0, *p* = 0.008, *r*_BS_ = 0.087) showed negligible effect sizes with overlapping quartiles. No differences were observed for SF‐36 pain or social aspects (adjusted *p* always > 0.119). Overall, the NP‐positive feature group consistently exhibited worse pain, reduced functional performance, and impaired general and mental health outcomes.

**TABLE 1:** Descriptive analysis and comparison between groups.

Variables	NP‐negative group (*N* = 644)			NP‐positive group (*N* = 258)			Adjusted *p*	Effect size	Overall results
	median	1Q	3Q	median	1Q	3Q			
Age (years)	69.7	64.0	75.5	69.1	64.8	75.0	0.661	0.019	NS
Knee range of motion (degrees)	95.0	90.0	110.0	95.0	90.0	100.0	0.289	0.048	NS
Visual Analogic Scale (score)	8.0	7.0	9.0	9.0	8.0	10.0	< 0.001	−0.223	↑ in NP_POS_
KSS, objective scores	42.0	30.8	52.0	40.0	28.0	49.8	0.119	0.072	NS
KSS, functional scores	40.0	30.0	55.0	40.0	21.2	50.0	0.171	0.062	NS
WOMAC, pain (score)	13.0	10.0	15.0	14.0	11.0	16.0	< 0.001	−0.155	↑ in NP_POS_
WOMAC, rigidity (score)	5.0	4.0	6.0	5.0	4.0	6.0	0.002	−0.142	↔
WOMAC, function (score)	48.0	39.0	55.0	51.0	44.0	59.0	< 0.001	−0.165	↑ in NP_POS_
WOMAC, total (score)	65.0	54.8	76.0	69.0	59.0	80.8	< 0.001	−0.171	↑ in NP_POS_
SF36, functional capacity (score)	10.0	5.0	20.0	10.0	0.0	20.0	0.028	0.099	↔
SF36, physical aspects (score)	0.0	0.0	0.0	0.0	0.0	0.0	0.008	0.087	↔
SF36, pain (score)	31.0	22.0	41.0	31.0	21.0	41.0	0.119	0.072	NS
SF36, general health status (score)	50.0	40.0	60.0	45.0	35.0	60.0	0.008	0.118	↓ in NP_POS_
SF36, vitality (score)	50.0	40.0	65.0	45.0	30.0	60.0	< 0.001	0.179	↓ in NP_POS_
SF36, social aspects (score)	50.0	25.0	62.5	50.0	28.1	62.5	0.474	0.032	NS
SF36, emotional aspects (score)	0.0	0.0	66.7	0.0	0.0	33.3	< 0.001	0.162	↓ in NP_POS_
SF36, mental health (score)	64.0	48.0	77.0	56.0	44.0	68.0	< 0.001	0.227	↓ in NP_POS_

*Note:* Data are presented as median (1st–3rd quartiles). Adjusted *p*‐values are from the Mann–Whitney *U* test with FDR correction for multiple comparisons. Effect size is calculated as the point‐biserial correlation coefficient (*r*_BS_). Overall results are interpreted as follows: NS, adjusted *p* > 0.05; ↑ or ↓ in NP_POS_, adjusted *p* < 0.05 with small‐to‐moderate effect size or a clear direction of differences; ↔, adjusted *p* < 0.05 but with negligible effect size or no clear direction of differences.

### 3.4 Associations Between Neuropathic Pain Features and Functional and Quality‐Of‐Life Outcomes

Multiple logistic regression was used to estimate the association between neuropathic pain features and functional and quality‐of‐life outcomes. Table 2 presents the set of variables associated with the occurrence of NP‐positive features. The model includes the WOMAC score and the social functioning, role‐emotional, and mental health domains of the SF‐36. The results indicate a positive association between NP‐positive features and WOMAC total score (OR = 1.018, 95% CI = 1.007–1.030, *p* = 0.001), as well as SF‐36 social functioning score (OR = 1.009, 95% CI = 1.002–1.016, *p* = 0.017). Conversely, NP‐positive features show a negative association with SF‐36 role‐emotional (OR = 0.995, 95% CI = 0.990–0.999, *p* = 0.011) and mental health (OR = 0.985, 95% CI = 0.977–0.994, *p* < 0.001) scores.

**TABLE 2:** Results of the multiple logistic regression analysis for factors associated with neuropathic pain (NP) signs.

Predictor variable	*β*	Odds ratio (95% CI)	*p* *
Intercept	−1.517	0.219 (0.078–0.615)	0.004
WOMAC, total (score)	0.018	1.018 (1.007–1.030)	0.001
SF36, social aspects (score)	0.009	1.009 (1.002–1.016)	0.017
SF36, emotional aspects (score)	−0.006	0.995 (0.990–0.999)	0.011
SF36, mental health (score)	−0.015	0.985 (0.977–0.994)	< 0.001

*Note:* Model notes: the dependent variable was the presence of neuropathic pain signs (coded as 1 if present, 0 if absent); the model included variables selected via backward stepwise elimination. CI, confidence interval; WOMAC, Western Ontario and McMaster Universities Osteoarthritis Index; SF‐36, 36‐Item Short Form Health Survey.

* *p*‐value obtained with the Walds' test. Model performance: AUC = 0.629; overall accuracy = 72.1%; sensitivity = 5.4%; specificity = 98.8%.

Regarding the model's overall performance, the area under the ROC curve (AUC) was 0.629, indicating limited discriminative ability. Using a probability cutoff of 0.5, the model demonstrated an overall accuracy of 72.1%; however, this figure is largely attributable to the high number of negative cases (specificity of 98.8%), as the model performed very poorly in identifying patients with neuropathic pain features (sensitivity of only 5.4%). Overall, the selected variables, although associated with NP‐positive features, lack sufficient predictive power for individual‐level screening.

## 4 Discussion

The aim of this study was to investigate the prevalence of NP features in patients with end‐stage KOA referred to TKA, and to explore its association with knee‐joint function and quality‐of‐life measures. Approximately one‐third of the patients exhibited NP, with a higher prevalence observed in female patients compared to their male counterparts. Knee functional performance and various dimensions of health‐related quality of life were negatively associated with the presence of NP features, with the WOMAC total score, as well as SF‐36 social functioning, role‐emotional, and mental health dimensions, being associated with DN4 positive signs in this population.

It is important to highlight that the main results of the study were based on the assessment of NP manifestation with the DN4 questionnaire. Although it is a widely used, valid questionnaire, the positive DN4 screening result attributed to the patients could be influenced by other components of pain (nociceptive, inflammatory, or mixed). Consequently, the primary results of this study must be interpreted with caution. It is also important to consider that, regarding the multivariate logistic model overall performance, the lower AUC and the unbalanced sensitivity/specificity results suggest that, although the identified variables are significantly associated with the presence of NP features, they do not collectively possess sufficient predictive power for individual clinical screening—a result consistent with the study's objective of investigating associated factors rather than developing a clinical prediction rule.

In the present study, 28.6% of patients with end‐stage KOA awaiting TKA exhibited signs of NP, as assessed by the DN4 questionnaire. The prevalence of NP in knee osteoarthritis varies considerably in the literature, ranging from 0.4% to 53% [7, 9, 10, 14]. This wide variation is attributed to factors such as knee osteoarthritis severity, treatment stage (e.g., outpatient versus presurgical), and the assessment method used [9, 25]. In fact, in reviews encompassing different stages, rates vary from 5.4% to 51.9% [9]. Studies focusing specifically on end‐stage patients, candidates for total knee arthroplasty, report prevalences between 2.5% and approximately 30% [14, 15], which aligns with the finding in the present study. High prevalences, close to 47% and 53%, have been observed in studies also using the DN4 in patients with painful primary KOA [7, 10]. Despite the variability, the current result is consistent with rates observed in other end‐stage cohorts. Furthermore, the higher prevalence in female patients (31.5%) compared to males (21.1%) confirms a robust trend reported in the majority of prior investigations [2, 3, 8, 12, 15], with the exception of one study [13].

The increased pain scores, regardless of the assessment tool used, are a prominent symptom in KOA patients with NP [2, 7, 12]. In terms of functional and quality‐of‐life characteristics, patients with positive signs of NP exhibited higher scores across nearly all dimensions of the WOMAC, along with lower scores in several SF‐36 dimensions, particularly general health, vitality, role‐emotional, and mental health (see Table 1). Overall, our results align with previous findings [2, 7, 8, 10, 11], although some differences may arise depending on the clinical status of the patient and the tool used to identify NP.

WOMAC scores are consistently higher in those with signs of NP, whether reported in outpatients with KOA [2] or in those with a painful [11], primary [10], or early‐stage KOA [7]. Interestingly, end‐stage KOA is associated with higher total WOMAC scores compared to outpatients with KOA, and total and pain WOMAC scores have been shown to rise with an increasing number of NP signs [14].

The specific association of NP features with WOMAC scores in early‐stage knee osteoarthritis varies significantly depending on the assessment tool used. The painDETECT questionnaire is associated with increased scores across the WOMAC pain, physical function, and total score domains. In contrast, identification via the DN4 tool correlates primarily with increases only in the WOMAC physical function domain [7]. Health‐related quality of life is also consistently poorer in patients with NP features, though the specific affected dimensions differ between studies. Research employing painDETECT shows declines in RAND‐36 physical functioning and role‐physical domains [8], as well as in SF‐36 bodily pain, social functioning, and role‐emotional domains [7]. Studies using the DN4 tool report a broader, albeit less consistent, influence, with findings ranging from no significant impact [12] to significant effects [2] across all SF‐36 domains, most notably in role‐physical, general health, and social functioning. Despite methodological variations in NP detection tools and patient cohorts, these findings align with observations in end‐stage disease. Clinically, this demonstrates that different screening instruments can reveal distinct profiles of patient impairment. Consequently, a positive result from either the painDETECT or DN4 questionnaire should trigger a more detailed clinical assessment, as it signals a complex pain state. The routine integration of such screening is a crucial step toward personalizing pain management in knee osteoarthritis.

Interestingly, while we found a clear association between NP features and the WOMAC, this was not observed for KSS. This discrepancy likely reflects the different constructs measured by these tools. The WOMAC captures the patient's subjective experience of pain and functional limitation, which can be markedly influenced by the central sensitization and psychological comorbidities (e.g., depression) [26] often associated with NP. In contrast, the KSS includes objective, surgeon‐assessed components of joint alignment and stability [27]. Consequently, a positive NP screen appears to be a marker of greater patient‐perceived disability and psychosocial burden preoperatively, rather than indicators of more severe structural joint pathology. This distinction underscores the complex, biopsychosocial nature of pain in end‐stage KOA and highlights the importance of assessing both pain mechanism and psychological state in addition to structural disease.

The most divergent findings of our study concerned the association between functional and quality‐of‐life measures and the presence of NP (Table 2). Previous investigations using various models, such as logistic and linear multiple equations, either did not assess the same measures (e.g., Güngör Demir et al. [2] tested variables such as symptom duration, depression score, and Kellgren–Lawrence class), or did not find any association with SF‐36 domains [12]. Using multiple logistic models, we found associations between the presence of NP and increases in WOMAC total and SF‐36 social functioning scores, as well as decreases in SF‐36 role‐emotional and mental health scores.

The contradictory finding was the positive association between NP and SF‐36 social functioning scores. While counterintuitive, this result may reflect a compensatory mechanism where patients with chronic NP search for greater social engagement for support, and it aligns with literature suggesting the social functioning domain is often less severely impacted by NP compared to other quality‐of‐life dimensions [28, 29]. Furthermore, this finding raises the intriguing possibility that stronger social support networks could themselves be associated with improved pain coping strategies, thereby contributing to better perceived social functioning (e.g., [30]). In this interpretation, the positive association might not merely reflect compensation for pain, but also a protective association against the detrimental correlates of pain in social life. In our model, controlling the strong negative associations with mental health may have isolated this relative resilience, making the positive statistical association more apparent, or it may reflect social activity maintained despite pain rather than due to its absence. This complex result highlights the multidimensional impact of NP. Nevertheless, given the cross‐sectional design and the multiple comparisons performed, this unexpected positive association should be interpreted with caution. It may also be influenced by residual confounding, statistical artifacts, or chance. Replication in independent cohorts is warranted before drawing firm conclusions.

### 4.1 Study Limitations

Several limitations of this study should be acknowledged. First, the uneven sex distribution—reflecting KOA epidemiology—limited analysis of sex‐specific effects. Second, we were unable to assess several confounding factors, including body mass index, depression (beyond the domain scores from SF‐36), analgesic medication usage, detailed comorbidity indices, validated psychological screening tools, osteoarthritis etiology and distribution, or socioeconomic data, as these variables were not systematically recorded in our electronic medical records and could not be retrospectively obtained. Although age was similar between groups, we cannot rule out residual confounding by age or other unmeasured clinical and psychosocial factors. Third and most importantly, NP was identified using the DN4 screening questionnaire, not a clinical diagnosis; some positive cases may have had other pain types. Despite this, the large sample size and extensive functional and quality‐of‐life characterization provide a robust profile of NP features in end‐stage KOA, identifying important clinical associations for future research to confirm. Concerning psychological factors, while we acknowledge the absence of dedicated psychological instruments, our SF‐36‐based assessment provides valuable proxy measures for mental health status of these patients, partially mitigating this limitation.

## 5 Conclusions

In summary, DN4‐positive neuropathic pain features affect roughly 29% of end‐stage knee osteoarthritis patients awaiting knee replacement, more often in females—this prevalence estimate should not be extrapolated to patients with early or moderate KOA, or to those not undergoing surgical evaluation. These features correlate with worse pain, function, and specific declines in mental health quality of life. These findings may highlight a potential need for preoperative pain phenotyping. Identifying these patients could allow for targeted strategies, as their distinct pain profile—particularly its psychosocial impact—could influence surgical outcomes. However, given the observational, cross‐sectional design of this study, these hypotheses require confirmation in prospective cohorts and interventional trials. Integrating a screening tool like the DN4 with psychosocial assessment could be considered as a basis for future pre‐habilitation protocols, such as patient education, neuropathic medication, and psychological support. A structured clinical pathway, with perioperative neuromodulatory intervention and postoperative vigilance for persistent pain, may be considered, though its effectiveness needs to be validated in randomized controlled studies before routine implementation.

## Author Contributions

**Alan P. Mozella:** conceptualization, writing – original draft, writing – review and editing. **Ana Carolina Leal:** conceptualization, formal analysis, writing – original draft, writing – review and editing. **Rafael Erthal de Paula:** data curation, writing – review and editing. **Aline Cordeiro Fernandes Ladeira:** data curation, writing – review and editing. **Sandra Minamoto:** data curation, writing – review and editing. **Matheus Carvalho:** data curation, writing – review and editing. **Jordanna Franco Sucupira:** data curation, writing – review and editing. **Ricardo Kobayashi:** data curation, writing – review and editing. **Thiago Lemos:** conceptualization, formal analysis, writing – original draft, writing – review and editing. Each author contributed individually and significantly to the development of this article.

## Funding

This study was funded by the Brazilian Ministry of Health, the Carlos Chagas Filho Foundation for Research Support of the State of Rio de Janeiro (FAPERJ), and the Coordination for the Improvement of Higher Education Personnel—Brazil (CAPES; Finance Code 001). A.C.L., A.C.F.L., and T.L. were recipients of FUNDACOR scholarships.

## Ethics Statement

The study was approved by the Ethical Committee of the Instituto Nacional de Traumatologia e Ortopedia—INTO (process no. 39785020.7.0000.5273, approved on 14 December 2020).

## Consent

We have obtained written informed consent from all the participants.

## Conflicts of Interest

The authors declare no conflicts of interest.

## Data Availability

The data that support the findings of this study are available upon reasonable request.
